# An investigation of data from the first year of the interim Canada Dental Benefit for children <12 years of age

**DOI:** 10.3389/froh.2023.1328491

**Published:** 2024-01-08

**Authors:** Robert J. Schroth, Vivianne Cruz de Jesus, Anil Menon, Olubukola O. Olatosi, Victor H. K. Lee, Katherine Yerex, Khalida Hai-Santiago, Daniella DeMaré

**Affiliations:** ^1^ Dr. Gerald Niznick College of Dentistry, Rady Faculty of Health Sciences, University of Manitoba, Winnipeg, MB, Canada; ^2^ Children’s Hospital Research Institute of Manitoba, Winnipeg, MB, Canada; ^3^ Shared Health Manitoba, Winnipeg, MB, Canada

**Keywords:** insurance, dental, child, health policy, public health dentistry, access to care

## Abstract

**Introduction:**

In 2022, the federal government announced a commitment of $5.3B to provide dental care for the uninsured, beginning with children <12 years of age. Now referred to as the Interim Canada Dental Benefit (CDB), the program targets those <12 years of age from families with annual incomes <$90,000 without private dental insurance. The purpose of this study was to review federal data from the Government of Canada on public uptake and applications made to the Canada Revenue Agency (CRA) during the first year of the Interim CDB.

**Methods:**

Data for the first year of the Interim CDB (up to June 30, 2023) were accessed from the Government of Canada Open Data Portal through Open Government Licence—Canada. Rates of children receiving the Interim CDB per 1,000 were calculated by dividing the number of beneficiaries by the total number of children 0–11 years by province or territory, available from Statistics Canada for the year 2021.

**Results:**

During the first year of the program, a total of 204,270 applications were approved, which were made by 188,510 unique applicants for 321,000 children <12 years of age. Over $197M was distributed by the CRA. Overall, the national rate for receiving the Interim CDB was 67.8/1,000 children. Ontario (82.5/1,000), Manitoba (77.1/1,000), Nova Scotia (73.4/1,000), and Saskatchewan (72.3%), all had rates of children with the Interim CDB above the national rate.

**Conclusions:**

Data from the first year of the Interim CDB suggests that this federal funding is increasing access to care for children <12 years by addressing the affordability of dental care. Governments and the oral health professions need to address other dimensions of access to care including accessibility, availability, accommodation, awareness, and acceptability of oral health care.

## 1 Introduction

The Canadian Academy of Health Sciences report, “Improving Access to Oral Health Care for Vulnerable People Living in Canada”, identified several groups facing considerable oral health disparities (1). Significant childhood oral health disparities exist (1–10), which is most evident among children from low-income homes, First Nations, Métis, and Inuit communities, refugee and immigrant groups, and rural and remote parts of Canada (5, 6, 9, 11–18). In the absence of a nationally coordinated approach to children's oral health in Canada, several provinces and territories either administer dental programs for, or provide dental benefits to, children from low-income households.

In March 2022, the federal government announced a commitment of $5.3B to provide dental care for the uninsured, beginning with children <12 years of age. Now referred to as the Interim Canada Dental Benefit (CDB), the program targets those <12 years of age from families with annual incomes <$90,000 without private dental insurance (19–23). Families must be Canadian citizens or permanent residents of Canada. Bill C-31, commonly referred to as the “cost of living relief for dental care and rental housing bill”, provides financial support up to $650 for each child if the family's adjusted net income is <$70,000, while $390 is provided if the family's adjusted net income is between $70,000 and $79,999, and $260 if it is between $80,000 and $89,999 (19–21). Nearly half a million children will benefit from this program.

To receive the Interim CDB, families must satisfy several requirements (24). This includes having a child or children <12 years of age at the time of application without access to private dental insurance, and an adjusted family net income <$90,000 per year. Additionally, families must have filed a 2022 tax return with Canada Revenue Agency (CRA) and incurred out-of-pocket dental care expenses for their child between October 1, 2022, and June 30, 2023 (24).

The federal government is presently developing a comprehensive Canadian Dental Care Plan (CDCP) for Canadians (25). Once fully implemented, the CDCP will support up to 9 million uninsured Canadians who have annual family net incomes <$90,000 (25). Budget 2023 proposed to provide $13B over 5 years and $4.4B ongoing to Health Canada to implement the CDCP (25). The CDCP is scheduled to begin at the end of 2023 for children up to 18 years, seniors, and those with special needs, with full implementation by 2025 for others (25). The CDCP is a significant first step in addressing access to dental care for Canadians as many people avoid seeking dental care because of the cost. However, it is essential to note that “affordability” is just one dimension of access to care (26). Others include accessibility, availability, accommodation, awareness, and acceptability (26).

The Interim CDB began in October 1, 2022 and will sunset June 30, 2024, while the CDCP is scheduled to commence by the end of 2023, providing coverage for uninsured Canadians with annual family income <$90,000 (24). The development of this national program provides tremendous opportunity for evaluation and ongoing policy development. The purpose of this study was to review federal data from the Government of Canada on public uptake and applications made to the Canada Revenue Agency (CRA) during the first year of the Interim CDB program.

## 2 Methods

This quantitative study involved evaluation of data from the CRA for national, provincial, and territorial trends in applications made to the Interim CDB. Data for the first year of the Interim CDB (up to June 30, 2023) were accessed from the Government of Canada Open Data Portal: https://open.canada.ca/data/en/dataset/a9e3f33b-f818-4e90-a936-01946fbe90f1, through Open Government Licence—Canada. The CRA regularly updates the benefit data on the Open Data Portal. Ethics approval was not required for this study as it involved aggregate, de-identified data that was publicly available from the federal government's Open Data Portal.

Specific variables under review included the number of applications submitted, number of unique applicants, number of children covered, and the total amount of funding provided for provinces and territories. Further, age grouping and gender of approved applicants, categories of adjusted family net income, ages of approved children, and amount approved by age grouping were available.

Available data were for all applications that were received as of June 30, 2023 and assessed as of July 7, 2023. Only applications with an approved amount are included in the tables. The number of applications and applicant province/territory of residence were obtained from the Canada Dental Benefit file. Province or territory was defined as the place of residence as of July 7, 2023. A unique applicant was defined as an individual, and applicants may have applied for more than one child. Applicants residing outside Canada were entitled to receive the Interim CDB if all eligibility criteria were met and these applicants were grouped in the “Outside Canada” category. All counts were rounded to the nearest ten and all amounts were rounded to the nearest thousand and were reported in thousands of dollars ($000). The sum of the data may not add to the total due to rounding, suppression, and/or double counting (for shared custody). A zero “0” indicates that the information has been suppressed for confidentiality purposes. Suppressed information also includes valid zeros. Applicants identifying as non-binary were categorized as gender diverse.

Rates of children with the Interim CDB per 1,000 were calculated by dividing the number of children with the benefit by the number of Canadians aged 0–11, by province or territory, based on census 2021 data available from Statistics Canada (27). Analysis included descriptive statistics (frequencies and proportions) done in Microsoft Excel.

## 3 Results

During the first year of the program, a total of 204,270 applications were approved, which were made by 188,510 unique applicants for 321,000 children <12 years of age (Table 1). Over $197M was distributed by the CRA, with the majority of funding going to applicants in the province of Ontario (46.0%). The greatest number of applications were from Ontario (44.5%) and the least from the territories [Northwest Territories (0.11%), Nunavut (0.6%), and Yukon (0.03%)]. When considering the gender of those filing applications with the CRA, the majority were made by females (178,810 of 188,510, or 94.9%). Only 9,160 of applications were made by males (4.9%). There were a total of 20 applicants identifying as being gender diverse. However, data for gender diverse applicants by provinces and territories were suppressed for confidentiality purposes. Based on the amount of money distributed in each province and territory in Canada, data showed that females were the predominant beneficiaries ($189,344,000 for female applicants and $7,723,000 for male applicants).

**Table 1:** Number of approved interim CDB applications, unique applicants, children and total amount distributed by province/territory.

Province/Territory	Number of applications	Number of unique applicants	Number of children	Rate of child participation (per 1,000 children)	Total amount ($000)
Newfoundland and Labrador	1,900	1,720	2,790	53.3	1,758
Prince Edward Island	520	470	780	42.6	470
Nova Scotia	5,070	4,590	7,880	73.4	4,989
New Brunswick	3,250	2,920	5,050	58.3	3,144
Quebec	37,830	34,250	57,400	52.2	33,639
Ontario	90,940	85,030	145,610	82.5	90,695
Manitoba	9,210	8,100	15,530	77.1	9,775
Saskatchewan	7,800	6,800	12,810	72.3	8,123
Alberta	24,140	22,340	39,120	60.9	23,862
British Columbia	23,190	21,910	34,230	60.9	20,712
Yukon	60	60	90	16.5	51
Northwest Territories	230	200	410	59.9	256
Nunavut	130	110	220	22.1	133
Outside Canada	10	10	20	–	11
Total	204,270	188,510	321,000	67.8	197,619

Table 1 also reveals that the majority of children receiving the Interim CDB were residents of Ontario (45.4%). However, to adjust for population differences among Canadian provinces and territories, rates of children with the Interim CDB, expressed per 1,000 children, were calculated using Statistics Canada population statistics for children aged 0–11. Overall, the national rate was 67.8/1,000 children. Ontario (82.5/1,000), Manitoba (77.1/1,000), Nova Scotia (73.4/1,000), and Saskatchewan (72.1%), all had rates of children with the Interim CDB above the national rate.

Table 1 also reports the total amount in funding that was distributed to applicants by province or territory. The largest amount of funding was distributed to residents of Ontario ($90,695M), which represented 45.9% of all federal funds disbursed. The least amount of funding went to the territories [Northwest Territories (0.13%), Nunavut (0.07%), and Yukon (<0.02%)].

Overall, 45.6% of applicants had a net adjusted family income <$30,000 (Table 2). Based on adjusted net family income thresholds established for the Interim CDB, most children received $650 towards their oral health expenses (91.1%) as their family's adjusted net income was <$70,000 (Table 2). Another 5.3% of children received $390 while 3.6% of children received $260.

**Table 2:** Number of approved interim Canada dental benefit applications, unique applicants, children and total amount (in $000) by adjusted net family income.

Adjusted family net income	Payment amount per child	Number of applications (%)	Number of applicants (%)	Number of children (%)	Total amount ($000)
Less than $10,000	$650	21,280 (10.4)	19,370 (10.3)	34,670 (10.8)	22,242
$10,000–$19,999	$650	32,150 (15.7)	29,060 (15.4)	51,650 (16.1)	33,228
$20,000–$29,999	$650	40,760 (20.0)	37,500 (19.9)	64,130 (20.0)	41,067
$30,000–$39,999	$650	33,080 (16.2)	30,610 (16.2)	51,710 (16.1)	33,026
$40,000–$49,999	$650	25,180 (12.3)	23,360 (12.4)	39,200 (12.2)	25,062
$50,000–$59,999	$650	19,000 (9.3)	17,710 (9.4)	29,580 (9.2)	18,923
$60,000–$69,999	$650	14,470 (7.1)	13,480 (7.2)	22,490 (7.0)	14,408
$70,000–$79,999	$390	10,900 (5.3)	10,320 (5.5)	16,960 (5.3)	6,618
$80,000–$89,999	$260	7,450 (3.6)	7,110 (3.8)	11,550 (3.6)	3,044
Total		204,270	188,510	321,000	197,619

**Figure 1:**
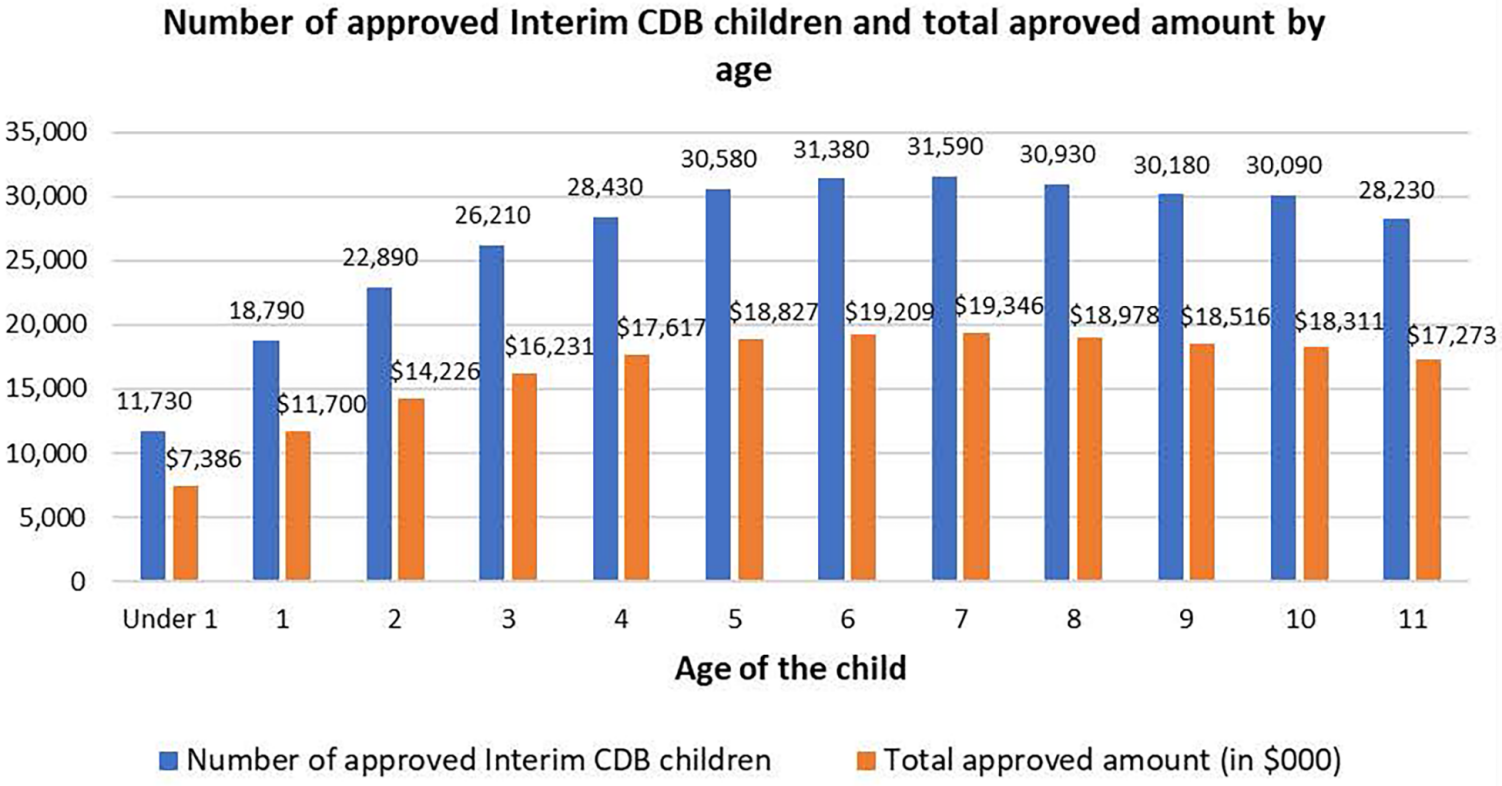
Total number of children approved for the Canada dental benefit (CDB) and total approved amount (in $000), by age.

The number of children by age grouping appears in Figure 1. Surprisingly, preschool children (those <6 years of age) accounted for 43.2% of children who received the Interim CDB, with >$85M of total approved benefits allocated to this age group. A total of $19,086M of approved benefits went to children ≤1 year of age. The highest amount of total approved benefits was in the 7-year-old group (>$19.3M).

Table 3 reports data on distribution of applicants by age group for the various provinces and territories. Most applicants were between the ages of 25 and 44 years (83.7%). This same pattern was true for all provinces and territories.

**Table 3:** Number of approved Canada dental benefit unique applicants by province and territory, and age group.

Province/Territory	Age group *N* (%)							Total
	<25	25–34	35–44	45–54	55–64	65+	Unknown	
Newfoundland and Labrador	110 (6.4)	790 (45.9)	680 (39.5)	120 (7.0)	20 (1.2)	0 (0.0)	0 (0.0)	1,720
Prince Edward Island	10 (2.1)	210 (44.7)	200 (42.6)	60 (12.8)	0 (0.0)	0 (0.0)	0 (0.0)	470
Nova Scotia	250 (5.5)	2,220 (48.4)	1,720 (37.5)	340 (7.4)	40 (0.9)	20 (0.4)	0 (0.0)	4,590
New Brunswick	140 (4.8)	1,410 (48.3)	1,110 (38.0)	230 (7.9)	10 (0.3)	0 (0.0)	0 (0.0)	2,920
Quebec	590 (1.7)	11,090 (32.4)	17,170 (50.1)	5,090 (14.9)	250 (0.7)	50 (0.2)	20 (0.1)	34,250
Ontario	2,300 (2.7)	31,100 (36.6)	40,280 (47.4)	10,370 (12.2)	710 (0.8)	180 (0.2)	100 (0.1)	85,030
Manitoba	440 (5.4)	3,460 (42.7)	3,410 (42.1)	680 (8.4)	80 (1.0)	20 (0.3)	0 (0.0)	8,100
Saskatchewan	550 (8.1)	3,040 (44.7)	2,580 (37.9)	510 (7.5)	90 (1.3)	20 (0.3)	0 (0.0)	6,800
Alberta	720 (3.2)	8,660 (38.8)	10,480 (46.9)	2,230 (10.0)	150 (0.7)	50 (0.2)	40 (0.2)	22,340
British Columbia	410 (1.9)	6,650 (30.4)	11,270 (51.4)	3,300 (15.1)	190 (0.9)	80 (0.4)	10 (0.1)	21,910
Yukon	0 (0.0)	20 (33.3)	30 (50.0)	0 (0.0)	0 (0.0)	0 (0.0)	0 (0.0)	60
Northwest Territories	10 (5.0)	120 (60)	60 (30.0)	0 (0.0)	0 (0.0)	0 (0.0)	0 (0.0)	200
Nunavut	20 (18.2)	60 (54.6)	30 (27.3)	0 (0.0)	0 (0.0)	0 (0.0)	0 (0.0)	110
Outside Canada	0 (0.0)	0 (0.0)	10 (100)	0 (0.00	0 (0.00	0 (0.0)	0 (0.0)	10
Total	5,550 (2.9)	68,830 (36.5)	89,010 (47.2)	22,950 (12.2)	1,550 (0.8)	440 (0.2)	190 (0.1)	188,510

## 4 Discussion

This evaluation of federal data from the Government of Canada Open Data Portal reveals that there has been considerable uptake by families for the Interim CDB and significant expenditures by the Canadian government to support dental costs for children <12 years of age from families with net incomes below $90,000. This is a positive development as the 2007–2009 Canadian Health Measures Survey revealed that nearly one-third of Canadians lack dental insurance (28). That report also revealed that 57% of children 6–11 years of age had dental caries, having on average 2.5 teeth affected by decay (28). Further, dental visits to address dental problems in children accounted for approximately 226 million lost school days (28).

Based on the available data, most families that applied for the Interim CDB in the first year (October 1, 2022 to June 30, 2023) of the program received the maximum eligible amount being offered ($650). For children in most jurisdictions of Canada this maximum yearly allotment would be sufficient to cover the costs associated with an examination, radiographs, cleaning, fluoride varnish application, and other preventive services such as dental sealants, but likely falls short of being enough to cover the costs of restorative procedures (i.e., fillings).

Considerable regional variation exists in the number of applications made, the number of children covered, and the amount of funding distributed. Also, females were the dominant beneficiaries, which could suggest a significant gender disparity in terms of program utilization and parental awareness of the benefit. Additionally, while Ontario, followed by Manitoba, had the highest number of applicants, Yukon, Nunavut, PEI and Quebec have much smaller numbers of applicants.

The low uptake in Canada's north cannot be ignored and may be related to the considerable access to care challenges in remotes parts of the country. While Medicare in Canada is the envy of many nations, the opposite is true for the accessibility and affordability of basic dental care for its most vulnerable populations, who generally have the most unmet needs (29). For instance, there is a documented shortage of providers and a lack of dental clinics in many remote northern communities. More importantly, the current application guidelines stipulate that applicants must identify the name and location of the dental provider along with the date of the child's scheduled appointment. This can be extremely challenging for many living in remote communities where there are no providers or dental clinics, which would discourage parents from even applying. The oral health literacy of parents may also influence applying for the program. The low uptake in the three northern territories might also be explained by many of these children having dental insurance through the federal Non-Insured Health Benefits program or Yukon's legislated children's dental public health program (30).

When considering rates of children receiving the Interim CDB, we get a better sense of the actual impact it may be having across Canada. For residents of provinces and territories that do not have legislated children's dental public health programming, such as Manitoba and Saskatchewan (30) the Interim CDB is a much needed support. Interestingly, rates of children with the Interim CDB were above the Canadian average in Manitoba along with Saskatchewan.

Good dental health during early childhood puts children on the proper foundation for a lifetime of optimal oral health. First dental visits before or no later than the first birthday have been recommended by several organizations (31, 32). Data reviewed as part of this study indicate that preschool aged children accounted for over 40% of all children who received the Interim CDB. It is encouraging that almost 10% of funded children were ≤1 year of age, which perhaps suggests that more parents are aware of the importance of early first dental visits. It might also be an indication that more dental offices are promoting the first dental visit for infants (33, 34). COVID-19 sharply reduced access to dental care for children in Canada and it also halted the capturing of oral health surveillance data for school-aged children in provinces and territories. This has resulted in a lack of evidence of the true burden of dental disease in the pediatric population.

The data in Table 2 reveals a lower participation rate for the Interim CDB among the higher income groups. This may be because they have less need for it compared to the lowest income groups covered. Additionally, these families may lack awareness about the benefits of the program, assuming that it is only meant for those with lower incomes. Moreover, the reduced benefits they receive through this program could make it less appealing to them.

The Interim CDB has generated considerable enthusiasm in Canada by pledging to enhance the affordability of dental care and empower Canadians to make autonomous healthcare decisions. However, the excitement surrounding the CDB's mission to seamlessly integrate oral health into overall healthcare is tempered by crucial considerations of potential challenges (35). These challenges include the introduction of an online application system through the CRA platform, potentially excluding individuals with limited digital access. Moreover, linking benefits primarily to family income, rather than clinical need, and maintaining open-ended dental service coverage pose challenges in ensuring equitable and consistent care. Distinguishing the Interim CDB from other programs, addressing disparities between public and private coverage, and tackling factors such as accessibility, affordability, availability, accommodation, acceptability, and awareness are crucial for the program's success. Policymakers must adopt a holistic approach, considering diverse needs and engaging stakeholders to navigate these challenges and fortify the Interim CDB's role in transforming dental care accessibility and patient autonomy in Canada. It is also essential to recognize the importance of considering input from oral health professionals, incorporating their evidence and opinions, to shape the future CDCP effectively. This approach ensures a comprehensive and informed strategy for integrating oral health into overall healthcare while empowering patients in their treatment choices (35). It is also important to note that children with private insurance are not eligible for the Interim benefit even though not all private insurance plans are robust and also leave many patients underinsured.

This study is not without limitations. While the Government of Canada Open Data Portal contains data on the number of applicants and the number of children insured, it does not include any details on the types of dental care the child received, nor does it provide insight into the actual oral health status of Canadian children. Data on the types of dental care services that were received by children approved as part of the Interim CDB are not available as it was a benefit directly paid to parents, rather than an insurance plan. The upcoming CDCP will likely make such data available. There was also no data on the gender of the children receiving the benefit, which prevented us from looking at any potential gender disparities with the Interim CDB. If possible, the federal government should attempt to track and monitor the gender of children receiving the benefit and in future receiving services through the Canadian Dental Care plan to ensure that there are no gender disparities as culture may influence oral health seeking behaviours of parents for their children. Fortunately, the current Canadian Health Measures Survey (Cycle 7) includes oral health assessments of Canadian children, including preschoolers, which will provide national estimates of oral health and disease. Since part of the CRA application process requires parents and caregivers to provide a date of visit and the name and address of their child's dental provider the likelihood of parents not seeking dental care for their insured children is low. Therefore, we have confidence that the majority of approved children have actually accessed some dental care during this first year of the Interim CDB.

## 5 Conclusions

Data from the first year of the Interim CDB suggests that the $197M in federal funding is increasing access to care for children <12 years of age in Canada as it helps address the affordability of dental care. While affordability is a major dimension of access to care, it is naïve to think that addressing financial barriers alone will allow Canadian children to reach their best oral health potential. Governments and the oral health professions need to ensure that services for children are also available in their communities, especially in rural and remote regions of Canada. They also need to address structural barriers to oral health care for children by tackling accessibility, accommodation, and availability of services where they reside. Furthermore, there must be ongoing efforts to address cultural and personal barriers to dental care by increasing the awareness and acceptability of oral health care to families and caregivers. While it is commendable that the government is looking at the preventive aspect of dental care there are many children who may still fall through the cracks as they, unfortunately, have developed considerable dental disease that requires immediate surgical intervention, such as severe early childhood caries.

## Data Availability

The original contributions presented in the study are included in the article/Supplementary Material, further inquiries can be directed to the corresponding author.
